# Unveiling Pakistan’s transport problems: a call to safeguard public health

**DOI:** 10.3389/fpubh.2024.1325193

**Published:** 2024-06-12

**Authors:** Ashna Habib, Tooba Ali, Zainab Nazir, Fiza Muskan, Ifra Jawed, Aymar Akilimali

**Affiliations:** ^1^Dow University of Health Sciences, Karachi, Pakistan; ^2^Dow International Medical College, Dow University of Health Sciences, Karachi, Pakistan; ^3^Medical Research Circle, Goma, Democratic Republic of Congo

**Keywords:** public transportation, developing countries, infectious diseases, road safety, policy measures

## Abstract

Public transportation is an important mode of transportation in developing countries like Pakistan since it is accessible and convenient. But there are also serious health hazards associated with it, especially when it comes to the transmission of infectious diseases including COVID-19, TB, and *Haemophilus influenzae*. Worldwide transportation systems are vulnerable, as the COVID-19 pandemic has shown, underscoring the necessity for study and mitigating measures. The danger of disease transmission is increased in Pakistan by crowded metropolitan areas, inadequate sanitation, and low health awareness. In addition, congested public transportation and inadequate ventilation lead to reduced air quality and elevated stress levels among commuters. Comprehensive actions are needed to address these health hazards, such as promoting physical distance, improving cleanliness, enforcing traffic safety laws, and implementing policy changes that support sustainable transportation. Community involvement and advocacy are critical in campaigning for safer and more sustainable transportation networks. Pakistan can enhance public health outcomes and reduce the health hazards linked to public transportation by giving priority to these measures.

## Introduction

In developing countries like Pakistan, public transport is becoming an increasingly important means of transportation since it is accessible and convenient for the public. But despite all its advantages, using public transportation poses a serious risk of spreading several infectious diseases, especially respiratory conditions including COVID-19, *Haemophilus influenzae*, and tuberculosis (TB). In addition to revealing weaknesses in international supply chains and transportation systems, the COVID-19 epidemic has caused unparalleled disruptions across the board. Researchers have been working intensively to evaluate the pandemic’s effects and develop mitigation and adaptation plans (1). The pandemic is posing unprecedented challenges to contemporary operations and supply chains (2), impacting not only the emergency response needs of communities, but also the logistics of handling vital supplies (3). Because respiratory droplets carrying diseases can stay in the air and infect passengers, public transportation provides an ideal environment for airborne infections. The danger of disease transmission is further increased by factors like crowded spaces, poor ventilation, and regularly touched objects like doorknobs and railings. As a result, prolonged exposure to these conditions has increased the risk of contracting meningococcal and tuberculosis (4), especially in areas of Pakistan that are heavily inhabited. Pakistan is particularly vulnerable to infectious disease epidemics due to several variables, including densely populated urban areas, poor sanitation, and socioeconomic inequality. The danger is also increased by low health awareness and inadequate immunization coverage. Therefore, it is critical to address the health risks related to public transportation in Pakistan. In addition to raising the danger of infectious disease transmission, overcrowded buses and trains significantly worsen air quality because of inadequate ventilation and vehicle emissions. The emotional and mental health problems caused by accidents and traffic jams also highlight how vital it is to implement comprehensive measures to protect the public’s health in transit systems.

## Associated health risks with public transportation

Although they are essential for urban mobility, public transit systems provide serious health concerns to commuters. Public transportation’s detrimental effects on health call for further research and preventative action, particularly in Pakistan. Table 1 summarizes the health risks associated with public transportation.

**Table 1:** Health risks associated with transportation.

Health risk	Associated factors	Example
Infectious Diseases	Population density, Close interaction among people	SARS, Avian Fli
Diarrheal Disorders	Overcrowded areas, Lack of Sanitation Infrastructure	
Lower Respiratory Infections	Crowded areas, Poor Ventilation	Pneumonia
Tuberculosis (TB)	Overcrowding, Close contact with infected individuals	*Mycobacterium tuberculosis*
Road Traffic Accidents	Unsafe road crossings, Lack of pedestrian safety measures	Pedestrian vehicle collisions
Stress Related Health Issues	Longer travel times, Lack of predictability, crowding	Sleep disturbances, Low self related health

### Infectious diseases and population density

Urban environments, with their high population density and strong interpersonal contact, have the potential to become hotspots for the fast spread of newly developing infectious diseases like the avian flu and the severe acute respiratory syndrome (SARS) (5). Containment environments like public transit, schools, and hospitals are ideal for the growth of airborne pathogens. Lower respiratory infections and diarrheal diseases continue to be major concerns, especially in low- and middle-income nations (6), which emphasizes the need for better sanitation and disease preventive strategies.

### Road safety challenges

In many nations, including Pakistan, road safety is still a major concern despite improvements in transportation infrastructure. Most road deaths and Road Traffic Injuries (RTIs)—more than 90% of them—occur in low- and middle-income (LMI) nations (7). Different categories apply to Pakistan’s road safety problems. Organizational, tactical, and strategic levels can be used to group them (7). Pedestrians, who are frequently disregarded by traffic laws, are mostly responsible for road accidents because of poor infrastructure and a lack of knowledge about safe crossing procedures. The danger of accidents is further increased by poorly designed work zones and poor road infrastructure, underscoring the significance of putting pedestrian safety first and following safety design requirements.

### Stress and mental health

In large cities, transportation stress is a common occurrence and can have several adverse impacts on one’s health and general wellbeing. Nevertheless, little is known about the stressors that are connected to it (8). Both staff and students experience higher levels of stress because of long commutes, erratic scheduling, and crowded classrooms. Transport-related stress can directly contribute to mood disorders (9) and emotional instability (10). Furthermore, extended periods of stress brought on by travel might impair subjective well-being (11). More importantly, transportation stress can cause negative spillover effects in other parts of life (12). Proactive steps must be taken to upgrade the transportation network, shorten commutes, and ease traffic to address these stresses.

## Factors associated with health concerns

Pakistan’s primary healthcare system confronts various obstacles, including emergency rooms that are ill-prepared to manage trauma cases and other hospitals that lack necessary equipment (13). In addition, the lack of adequately furnished emergency medical facilities and skilled pre-hospital staff causes delays in the delivery of emergency care (14). These difficulties are further compounded by hospital trauma treatment capacity and coordination deficiencies, which impede prompt diagnosis and treatment of injuries sustained in traffic accidents (15). Moreover, Pakistan does not have formalized protocols in place for routinely gathering data on injuries, noise and air pollution, and lead poisoning. Although programs supporting liquefied petroleum gas and compressed natural gas are being implemented to lower vehicle emissions, catalytic converters are still not widely used (16). Positively, the Punjab government (a province of Pakistan) has improved the infrastructure for urban transportation by constructing bike lanes on important metropolitan routes and implementing comfortable bus services in Lahore, one of Pakistan’s largest cities (16). Unfortunately, the environmental effects of motor vehicles, such as the emission of pollutants like carbon dioxide and black carbon, exacerbate urban heat island effects and contribute to global warming, which can have a negative influence on health and result in early death and cardiorespiratory issues (17).Global warming and climate change are linked to an increase in common diseases, with an estimated 150,000 to 250,000 premature deaths related to human-caused climate change each year (18). Addressing these complex health challenges necessitates comprehensive strategies that include healthcare infrastructure improvements, pollution control measures, and sustainable mobility initiatives.

### The critical need for action

Urgent actions are necessary to address the complex difficulties arising from Pakistan’s transportation landscape, given the unbelievable toll that the country’s transportation system is having on public health. Every year, a lack of physical activity results in the premature death of over 2.1 million people. This problem is made worse by the fact that driving is a sedentary activity and there are few opportunities for active commuting. The risk of serious health issues such as diabetes, dementia, cardiovascular diseases, breast cancer, and colon cancer increases with this sedentary lifestyle (19). Every year, road motor vehicle accidents claim the lives of about 1.5 million people worldwide and injure 79.6 million more (20). The sneaky air pollution, which comes from burning tires and vehicle emissions, causes chronic bronchitis, aggravates adult chronic obstructive pulmonary disease, and aggravates pediatric asthma (21). It also plays a major role in the increase of greenhouse gas emissions. Lead poisoning, which has a significant negative impact on children’s behavior, hearing, and IQ, is caused by the usage of lead gasoline, which exacerbates the problem (22). A growing number of diseases linked to motorized mobility necessitates unwavering focus and swift action as data links harmful health impacts to environmental exposures and lifestyle factors. The need is obvious: Pakistan’s transportation problems are closely linked to harmful health effects and environmental consequences that must be addressed with a thorough and prompt response.

## Potential solutions

Pakistan’s transportation problems call for an integrated approach that incorporates actions based on solid facts and customized to the nation’s particular circumstances. Prioritizing cost-effective measures including healthcare worker training, quality improvement approaches, needs assessments, and optimal resource allocation can be achieved by drawing on the research on integrated trauma care systems in low- and middle-income countries (LMICs) (23). However, these strategies must be tailored to the unique problems and dynamics of the Pakistani context.

### Infrastructure improvements

Comprehensive infrastructure upgrades should be given top priority by transportation authorities to increase accessibility and safety for all users of the road. Adding more, segregated lanes exclusively for motorcyclists, cyclists, and pedestrians is one practical strategy. Marshall et al. proposed that enhancing bike infrastructure with more protected/separated cycling facilities is significantly associated with fewer fatalities and improved road safety outcomes for all road users (24). Additionally, spending money to upgrade road features like smoother surfaces and improved signs can greatly increase safety. The term “traffic calming” refers to techniques used in road design to lower vehicle volumes and speeds (25). In metropolitan settings, traffic calming measures are required to lower traffic volumes and speeds as well as the likelihood that pedestrians engaged in fatalities may die (26). Enough lighting is also necessary for pedestrian walkways and roadways, especially at night or in poorly lit regions, to enhance visibility and lower the chance of accidents.

### Promotion of physical distancing

Putting policies in place to encourage physical distance in public transportation environments can aid in lowering the risk of disease transmission. This may involve establishing an upper limit on the number of passengers, designating certain zones for seating to guarantee enough room, and varying the timing of commutes to reduce congestion during rush hours.

### Enhanced sanitation and hygiene measures

Reducing the spread of infectious diseases requires the implementation of thorough sanitation and hygiene practices. To reduce the transmission of infections, this involves routinely disinfecting stations, buildings, and vehicles used by public transportation. Furthermore, preventing the spread of gastrointestinal and respiratory diseases can be achieved by giving commuters access to hand sanitizers and encouraging good hand hygiene practices through awareness programs.

### Enforcement and education

Road safety is a complicated process that is mostly dependent on human factors in addition to technological and environmental advancements (27). It is believed by scientists that human behavior accounts for between 70 and 80% of traffic accidents and injuries (27). Because of this, it is critical to comprehend how drivers interact with traffic laws, enforcement, penalties, and justice in general (27). To guarantee that traffic laws are followed and to discourage risky driving, transportation authorities should concentrate on bolstering their enforcement systems. This involves putting in place thorough vehicle safety inspections to find and fix technical problems that can jeopardize traffic safety. Programs for driver education should also be implemented to raise motorists’ understanding of safe driving techniques and encourage responsible behavior. Large-scale public education efforts about road safety should be started at the same time, focusing on both rural and urban areas. Adhering to traffic regulations, abstaining from unsafe driving practices, and respecting the rights of other road users should be the main messages of these campaigns.

### Policy measures

Policy interventions are critical for resolving structural issues and promoting a culture of road safety. Muhlrad et al. (28) specifies six components of road safety policies, which are the result of policy design and policy acceptance and will influence implementation: (1) A long-term plan, ideally approved by the legislature and signed into law; (2) A medium-term plan that establishes the foundation for the development and execution of subsequent road safety intervention initiatives; (3) Quantitative short- to medium-term (four to five-year) goals for injury reduction that will be utilized to gage additional efforts; (4) A program for road safety that organizes all actions intended to reach the goals; (5) Establishing implementation conditions to guarantee that financial, technical, and human resources are accessible when needed; (6) A funding system guaranteeing yearly financing of the action program and support operations (28).

### Promotion of sustainable transportation

Encouraging environmentally friendly modes of transportation is essential to cutting down on harmful emissions and lessening the consequences of climate change. Reducing climate change requires the use of sustainable transportation technologies (29). Transportation authorities must give top priority to programs that promote the use of alternative fuel vehicles, like natural gas and battery electric cars. A variety of legislative actions, such as raising fuel quality requirements to lower emissions and tightening pollution regulations for new cars, can be taken to accomplish this. It is also crucial to promote walking, bicycling, and public transportation as practical substitutes for driving a private vehicle to lessen dependency on fossil fuels and cut emissions overall.

### Community involvement and advocacy

Local communities are the foundation of any effective advocacy campaign. Communities can express their needs, wants, and preferences for public transit when they band together. Residents can express their opinions and concerns via public forums, polls, and community gatherings, which can subsequently be used to inform advocacy campaigns. NGOs offer an “arm’s length” decision-making method, avoiding issues related to government bureaucracies. In Pakistan, NGOs have been successful in expanding public transit services, while private minibus operators work with the Faisalabad Urban Transport Society (FUTS) (30). Successful public transit programs have been created in countries like Hong Kong, Singapore, Australia, and Luxembourg, focusing on technological innovations, accountability, and transparency. These programs aim to enhance public transit accessibility, sustainability, and effectiveness while promoting more environmentally friendly urban mobility. Further analysis is needed to determine their long-term viability and potential for replication.

## Limitations and strengths

This perspective has certain limitations, despite the fact that it provides insightful information on health risks connected to public transportation in Pakistan and suggests viable solutions to these issues. One drawback is the absence of comprehensive data or Pakistan-specific case studies, which might offer additional background and proof to back up the suggested modifications. Furthermore, a perspective mostly concentrates on urban areas and does not adequately represent the health problems associated with transportation that Pakistani rural populations experience. A more thorough examination of the socioeconomic variables influencing the nation’s transportation-related health inequities would also be beneficial to the perspective.

Despite these drawbacks, this perspective offers an in-depth overview of the interrelated problems pertaining to health and public transportation in Pakistan. It draws attention to how urgent it is to address these issues and provides legislators, transit authorities, and public health professionals with practical suggestions. In overall terms, although there exists need for more study and enhancement, this perspective provides a useful foundation for tackling the intricate relationship between public health and transportation in Pakistan.

## Conclusion

The transportation sector in Pakistan is plagued by several problems that make it difficult for individuals to travel and get around, as well as many health-related problems. The current situation calls for improved infrastructure that can handle the increasing number of automobiles. For lower-income populations, the current transportation infrastructure is unavailable and too expensive. To lessen lengthy drives for work, school, or everyday activities, government policymakers must refocus their objectives. Additionally, Pakistan’s transportation system must construct its framework for sustainable mobility by taking lessons from advanced countries. The risk of infectious disease transmission, poor air quality from car emissions, and inadequate ventilation in public transportation are all growing concerns in Pakistan, making it imperative to address these issues.

## Data availability statement

The original contributions presented in the study are included in the article/supplementary material, further inquiries can be directed to the corresponding author.

## Author contributions

AH: Conceptualization, Supervision, Visualization, Writing – original draft, Writing – review & editing. TA: Conceptualization, Data curation, Writing – original draft, Writing – review & editing. ZN: Conceptualization, Methodology, Writing – original draft, Writing – review & editing. FM: Data curation, Writing – original draft, Writing – review & editing. IJ: Formal analysis, Writing – original draft, Writing – review & editing. AA: Validation, Writing – original draft, Writing – review & editing.
